# Biology and genetic diversity of *Candida krusei* isolates from fermented vegetables and clinical samples in China

**DOI:** 10.1080/21505594.2024.2411543

**Published:** 2024-10-02

**Authors:** Tianhong Zheng, Lingyu Ji, Yi Chen, Chengjun Cao, Jian Bing, Tianren Hu, Qiushi Zheng, Dan Wu, Haiqing Chu, Guanghua Huang

**Affiliations:** a Shanghai Institute of Infectious Disease and Biosecurity, Department of Infectious Diseases, Shanghai Key Laboratory of Infectious Diseases and Biosafety Emergency Response, National Medical Center for Infectious Diseases, Huashan Hospital, State Key Laboratory of Genetic Engineering, School of Life Science, Fudan University, Shanghai, China; b Shanghai Engineering Research Center of Industrial Microorganisms, Shanghai, China; c The International Peace Maternal and Child Health Hospital, School of Medicine, Shanghai Jiao Tong University, Shanghai, China; d College of Pharmaceutical Sciences, Southwest University, Chongqing, China; e Department of Respiratory and Critical Care Medicine, Shanghai Pulmonary Hospital, School of Medicine, Tongji University, Shanghai, China; f Shanghai Key Laboratory of Tuberculosis, Shanghai Pulmonary Hospital, School of Medicine, Tongji University, Shanghai, China

**Keywords:** *Candida krusei*, genetic diversity, population genetics, fermented food, antifungal resistance, ploidy variation

## Abstract

*Candida krusei*, also known as *Pichia kudriavzevii*, is an emerging non-*albicans Candida* (NAC) species causing both superficial and deep-seated infections in humans. This fungal pathogen is inherently resistant to the first-line antifungal drug, fluconazole, and is widely distributed in natural environments such as soil, foods, vegetables, and fruits. In this study, we collected 86 *C. krusei* strains from clinical settings and traditional fermented vegetables from different areas of China. Compared to *C. krusei* strains from fermented vegetables, clinical isolates exhibited a higher ability to undergo filamentation and biofilm development, which could facilitate its host colonization and infections. Isolates from fermented vegetables showed higher resistance to several antifungal drugs including fluconazole, voriconazole, itraconazole, amphotericin B, and caspofungin, than clinical strains, while they were more susceptible to posaconazole than clinical strains. Although *C. krusei* has been thought to be a diploid organism, we found that one-fourth of clinical strains and the majority of isolates from fermented vegetables (87.5%) are triploid. Whole-genome sequencing and population genetic analyses demonstrated that isolates from clinical settings and fermented food are genetically associated, and distributed across a wide range of genetic clusters. Additionally, we found that six nucleotide substitutions at the promoter region of the *ABC11* gene, encoding a multidrug efflux pump, could play a critical role in antifungal resistance in this species. Given the ubiquitous distribution of *C. krusei* strains in fermented vegetables and their genetic association with clinical strains, a One Health approach will be necessary to control the prevalence of this pathogen.

## Introduction

Fungal infections pose a significant global threat to human health [1]. *Candida* species are major agents causing both superficial and life-threatening systemic candidiasis [2–4]. While *Candida albicans* remains the predominant fungal pathogen in clinical settings, there has been an epidemiological shift towards non-*albicans Candida* (NAC) species in the last two decades [5]. Among these NAC species, *Candida krusei* has garnered special attention due to its increasing incidence of infection and intrinsic resistance to the first-line antifungal drug fluconazole [6]. Unlike the human commensal species *C. albicans* and *Candida glabrata*, *C. krusei* not only colonizes the human host as a commensal microbe but also widely distributes in environmental niches, including soil, vegetables, fruits, and fermented foods [6–8]. This fungus is also an important microorganism used in the fermentation process of beverages and industrial products [6,9,10]. A recent genomic and phylogenetic study demonstrates that the *Pichia kudriavzevii* isolates used in the industry are the same species as medically isolated *C. krusei* strains [11].

*C. krusei* belongs to the genus *Pichia* in the family *Pichiaceae* and is not a member of the traditional CTG *Candida* clade, the constituent species of which translate the CTG codon as serine instead of leucine [11,12]. Despite the phylogenetic distance from other pathogenic *Candida* species, *C. krusei* is able to cause a number of superficial and invasive infections, including vaginitis, oral thrush, endophthalmitis, onycholysis, endocarditis, osteomyelitis, and candidemia [6]. Previous studies have demonstrated that *C. krusei* exhibits the ability to undergo morphological changes, develop biofilms, and secrete aspartyl proteinases, which are critical for its adaptation to the host and its ability to cause infections [6].

Although *C. krusei* is less frequently isolated in clinical settings than *C. albicans* and *C. glabrata*, it attracts considerable attention due to its inherent resistance to several azole antifungal drugs and reduced susceptibility to flucytosine and amphotericin B [6,13]. It has been proposed that the widespread use of azole antifungal drugs, especially for prophylaxis purposes, possibly leads to the increased occurrence of *C. krusei* infections [14,15]. However, some multicenter trials report discrepant results [16,17]. The decreased drug affinity for *C. krusei* Erg11 and upregulated and constitutive expression of Abc1 and Abc11 efflux pumps could underlie the mechanism of its azole resistance [18–20]. However, compared to *C. albicans* and *C. glabrata*, the biology, genetic basis, pathogenesis, and antifungal resistance are still poorly understood.

Given its wide distribution in the environment, vegetables, fruits, and fermented foods, from a One Health perspective, these non-clinical niches could shape the genetic and biological features of *C. krusei*. Potential transmissions of this pathogen between environmental niches and clinical settings could occur due to human activities. In this study, we collected 86 *C. krusei* strains from traditional Chinese fermented vegetables (32) and clinical samples (54). We aimed to conduct a comparative investigation of the biological and genomic characteristics of these isolates from environmental and clinical sources. Based on these analyses, we sought to explore the potential environmental origin of antifungal resistance of clinical *C. krusei* isolates and establish the genetic association between environmental and clinical sources.

## Materials and methods

### Strain collection and culture conditions

All strains used in this study are listed in Dataset S1. Yeast extract-peptone-dextrose (YPD) agar plates (20 g/L peptone, 10 g/L yeast extract, 20 g/L glucose) were used for the routine growth of fungal cells. Clinical *C. krusei* strains were collected from the microbial resource collections of five Chinese hospitals. These isolates were originally isolated from sputum samples (23), vagina (10), urine (8), ascites (2), bile (2), blood (2), excreta (2), and other tissues. The detailed information for clinical strains are presented in **Dataset S1**. Environmental *C. krusei* strains were isolated from yeast species from fermented vegetables originally grown on YPD medium. Yeast cells of all strains were diluted and plated onto YPD agar plates. Single colonies were then picked and identified using matrix assisted laser desorption ionization – time of flight MS (MALDI – TOF MS) assays [21]. The *C. krusei* isolates were then verified by sequencing the nuclear ribosomal DNA internal transcribed spacer (ITS) [22]. Single colonies of each strain were subject to molecular identification and whole-genome sequencing. For morphological assays, fungal cells were grown on YPD agar plates supplemented with 5 μg/mL phloxine B (a red dye) and incubated at 30°C.

### Biofilm assays

Biofilm assays were performed according to a previous study with slight modifications [23]. Briefly, *C. krusei* cells were initially inoculated on YPD plates and incubated at 30°C for 2 days. The fungal cells were then adjusted to a concentration of OD_600_ = 0.5 in RPMI 1640 medium and transferred to 96-well cell culture plates for biofilm development. The plates were incubated in an incubator with shaking (250 rpm) at 37°C for 90 min. Subsequently, the wells were washed with 1 × PBS, replenished with fresh RPMI 1640 medium, and then incubated with shaking (250 rpm) at 37°C for 24 h. The medium was removed, and cell density of each well was assessed using a Tecan SPARK CYTO400 multimode microplate reader through Sparkcontrol V2.3 program. In the “Plate” page of the program, the plate type was set as “[COS96ft] - Costar 96 Flat Transparent,” “No lid,” “No humidity cassette.” In the “Absorbance” page, the measurement wavelength was set to 600 nm. For multiple reads per well, the size was set as 4 × 4 and the border set to 500 μm. The default values were used for all other settings. Three biological replicates were performed.

### Minimal inhibitory concentration (MIC) testing

Antifungal susceptibility of each strain was examined according to the CLSI document M27-A3 and previous descriptions [24]. The antifungal drugs were diluted in RPMI 1640 medium in 96-well plates. The ranges of antifungal drug concentrations were 1 mg/L to 512 mg/L for fluconazole, 0.0625 mg/L to 32 mg/L for amphotericin B, and 0.03125 mg/L to 16 mg/L for caspofungin, itraconazole, voriconazole, and posaconazole, respectively. Yeast cells were counted using a hemocytometer under the microscope. Approximately 500 cells were added into 200 μL RPMI 1640 medium and incubated at 35°C for 24 h. *Candida parapsilosis* ATCC 22019 and *Candida krusei* ATCC 6258 were used for quality control. Based on the Khalifa’s study and CLSI-M59, the epidemiological cut-off values (ECVs) for *C. krusei* (2 mg/L, 0.5 mg/L, 0.5 mg/L, and 1 mg/L, for AMB, POC, VOC, and ITC, respectively) were adopted [25,26]. In this study, minimal inhibitory concentration (MIC) represents the lowest drug concentration that inhibits 50% of fungal cell growth. Strains with an MIC value above the ECV were regarded as non-wild type (non-WT). Resistance to CAS and VOC was reported when the MIC values were ≥ 1 mg/L and ≥ 2 mg/L, respectively [27]. Due to the intrinsic resistance of *C. krusei* to fluconazole, we defined breakpoints for fluconazole as “relatively resistant (MIC value ≥ 64 mg/L)” and “relatively sensitive (MIC value ≤ 32 mg/L)” based on previous studies and resistance characteristics of the strains we collected [11].

### Cell ploidy analysis

Flow cytometry assays were initially performed to determine the ploidy of *C. krusei* strains as described in our previous study [28]. *C. krusei* cells were initially grown on YPD medium for 48 h. Then, cells were inoculated and incubated in a liquid YPD medium for overnight growth at 30°C. Cells were harvested, washed, and resuspended in 1 × TE buffer (10 mM Tris and 1 mM EDTA, adjusted pH to 8.0). Then, 700 µL of ethanol was mixed with 300 µL of fungal suspension. The mixture was incubated at room temperature for 2 h. Fungal cells were collected and washed with 1 × TE buffer and treated with RNase A (0.3 mg/mL) overnight, followed by proteinase K treatment (0.3 mg/mL) for 2 h at 50°C. The fungal cells were washed with 1 × TE buffer and stained with propidium iodide (PI, 0.025 mg/mL) for DNA content analysis.

The ploidy of all strains was reconfirmed by detecting the frequency of the non-reference allele for cumulative heterozygous biallelic SNPs across all scaffolds [29]. Heterozygous biallelic SNPs were extracted from VCF files using GATK SelectVariants with parameters “−selectType SNP” and “−restrictAllelesTo BIALLELIC.” The allele frequency was calculated as allele depth (AD)/read depth (DP) through a Python script. Aneuploidy was analyzed based on the relative copy number variations of chromosomes [30]. In brief, the read depth obtained with 1000-bp non-overlapping windows across the genome was generated with default parameters. The relative copy number values of each window were normalized to the median value of non-overlapping 1000-bp windows and visualized using an R script.

### Mutation, phylogenetic, and population genetics analyses

The genomic DNA of each *C. krusei* strain was extracted, and second-generation whole-genome sequencing assays were performed. Briefly, single colonies from each strain were picked, inoculated into liquid YPD medium, and cultured at 30°C with shaking at 220 rpm for 24 h. Fungal cells were then collected and genomic DNA was extracted using the TIANamp Yeast DNA Kit (TianGen Biotech, Beijing, China) according to the manufacturer’s protocol. Whole-genome sequencing (WGS) was performed using the PE150 sequencing and DNBseq tech platform (BGISEQ). For each strain, at least 3 GB of clean data was generated for subsequent analysis. The clean reads of each strain were aligned to the reference genome of strain CBS573 (Acc. No.: ASM305444v1) using the mem module of BWA v0.7.17 [31]. SNP and INDEL calling was performed using SAMTools v1.361 and Picard Tools v1.56 (http://picard.source-forge.net) and Genome Analysis Toolkit (GATK) v2.7.2. Reference-based alignment, variant calling, and calculation of the total number of SNPs were carried out using custom in-house scripts. Following parameters were used for the above programs: the maximum number of reads for calling an SNP = 10,000; the minimum mapping quality = 25; and the minimum base quality to identify putative SNPs = 25. The parameters for GATK program were: “stand_call_conf 50” “stand_emit_conf 20” “min_base_quality_score 25.” The variation sites with a coverage depth ≥15 were remained for subsequent analyses and final SNP extraction. Phylogenetic trees were constructed based on whole-genome SNPs, using RAxML v8.2.12 with GTRMMA model and bootstrap resampling was set to 1000 [32]. The visualization and colorstrips attachment were added to the phylogenetic tree using the ITOL web version [33]. Population structure analysis was conducted using ADMIXTURE v1.3.0 program, and the best-fit K value was determined through the program’s cross-validation (CV) procedure by selecting the K value with the lowest CV error [34]. Variscan v2.0.4 was used for population genomic analyses such as genome-wide nucleotide diversity (π) and Tajima’s D. The estimate of fixation index (F_*ST*_) between pairwise groups was calculated through genome-wide nonoverlapping windows of 10 kb using VCFtools v0.1.16 and visualized by an R script. The linkage disequilibrium decay was indicated by the mean value of the correlation coefficient (r^2^) through PLINK v1.9.

### Quantitative real-time PCR (qRT-PCR) assays

qRT-PCR assays were performed according to our previous publication [35]. Total RNA was extracted and reverse transcribed to cDNA using the GeneJET RNA Purification Kit and RevertAid H Minus reverse transcriptase (Thermo Scientific, Inc., Beijing, China). qRT-PCR analysis was performed using the Bio-Rad CFX96 real-time PCR detection system. The relative expression level of each gene was normalized by the *C. krusei ACT1* gene. Primer used are listed in **Dataset S2.**

### Introduction of nucleotide substitutions into the ABC11 promoter region of strain PK-34

The upstream and downstream flanking regions of the *ABC11* promoter were amplified from genomic DNA of strain PK-27, which contained the six nucleotide substitutions and was highly fluconazole-resistant. The positive selection marker gene *caSAT1* was amplified from plasmid pSFS2a [36]. The promoter region of *C. krusei ACT1* was amplified from genomic DNA of strain PK-34 and used as the promoter of *caSAT1*. A fusion PCR product was generated with these four fragments and transformed into strain PK-34. The transformants were verified through PCR and DNA sequencing assays. Primers used were listed in **Dataset S2**.

## Results

### Collection of *Candida krusei* isolates from fermented vegetables and clinical sources

As mentioned, the opportunistic human fungal pathogen *C. krusei* is widely distributed in fermented foods. In this study, we investigated the diversity of and genetic association between *C. krusei* isolates from fermented foods and clinical sources, as well as the possibility of environmental origination of its antifungal resistance. We collected a total of 86 *C. krusei* isolates, including 54 from clinical sources from five hospitals and 32 from fermented foods from seven farmer markets in China. The clinical strains were isolated from vaginal infections (*n* = 10), sputum samples or the oral cavity (*n* = 24), urine samples (*n* = 8), and deep-seated infections (*n* = 12, including samples from blood, pancreas, bile, ascites, and abdominal drainage fluid and secretions). Except for strains HS68–1 and HS68–2, which were isolated from the same patient, all other clinical strains were from independent patients. Fermented foods included Chinese mustard, fermented mooli, bamboo shoots, Chinese pickles, bell peppers, and sauerkraut. Detailed strain information is presented in supplementary Dataset S1.

### Filamentous growth and biofilm development of *C. krusei* isolates from fermented foods and clinical sources

**Figure 1.**
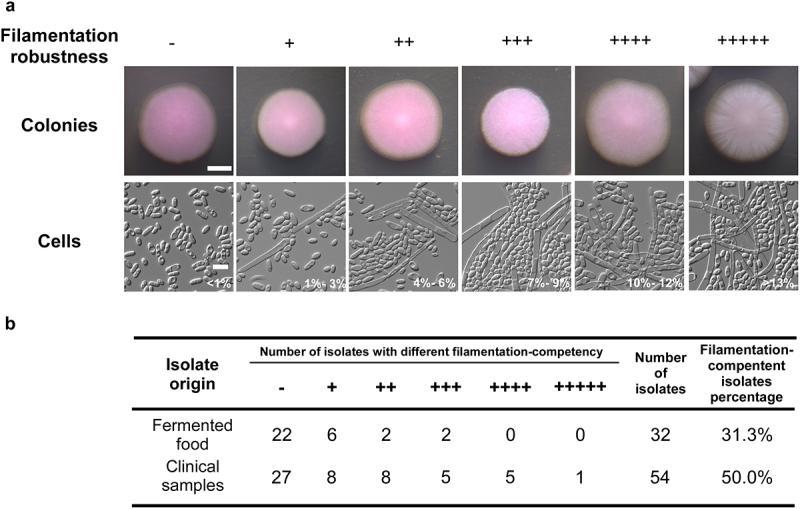
Filamentation-competency of *C. krusei* strains isolated from clinical samples and fermented foods. (a) Representative colony and cellular images of strains with different filamentation-competency. Approximately 150 cells were plated on YPD agar plates containing 5 µg/mL phloxine B and incubated at 30°C. The images of colonies were taken after 2 days of incubation and cellular images were obtained after 5 days of incubation. Scale bar for colonies, 4 mm; for cells, 10 μm. (b) Number of isolates with different filamentation-competency. Clinical samples: 24 from sputum samples or oral cavity, 10 from vaginal infections, 8 from urine samples, and 12 from deep-seated infections (blood, bile, abdominal drainage fluid, internal excreta, and ascites). The number of “+” symbols indicates the robustness of filamentation. “-” denotes no filamentous growth. Detailed information on filamentation for each strain is presented in dataset S1.

The ability of filamentous growth and biofilm development is critical for the virulence of pathogenic fungal species [37–41]. We first examined the ability of filamentous growth of all 86 *C. krusei* isolates on YPD medium at 30°C and 37°C. Generally, *C. krusei* strains exhibited a similar ability to develop filaments at the two temperatures. As shown in Figure 1a, we classified the strains into six groups based on colony and cellular morphologies and robustness of filamentation. 50.0% (27/54) of clinical strains showed the ability of filamentation (ranging from “+” to “+++++”), whereas only 31.3% (10/32) of isolates from fermented foods were able to develop filaments (ranging from “+” to “+++,” Figure 1b). Compared to the fermented food isolates, clinical *C. krusei* strains showed a generally stronger ability of filamentation based on robustness and percentage of filamentation-competent isolates (*p* = 8.23e − 03), suggesting that this ability of morphological change could be a strategy of adaptation to the host niche. The colony and cellular morphologies of 12 representative isolates are presented in Figure S1a and a detailed description of filamentation ability of all 86 strains is presented in supplementary **Dataset S1**.

We next examined the ability of biofilm formation of *C. krusei* isolates and did not find a significant difference between clinical strains and isolates from fermented food sources (Figures S1b and S2). However, a subset of the strains from vaginal infections, urine samples, and deep-seated infections exhibited a relatively higher ability of biofilm formation (Figure S2), which could contribute to the enhanced ability of tissue colonization and recurrent infections.

### Antifungal susceptibility of *C. krusei* isolates from fermented vegetables and clinical sources

Given the wide distribution of *C. krusei* in the environment and fermented food, the use of fungicides in agriculture and food preservation could potentially cause antifungal resistance. We compared the antifungal susceptibilities of *C. krusei* isolates from fermented foods and clinical sources (Table 1 and Table S1). Three major classes of antifungal drugs, including azoles (fluconazole-FLU, voriconazole-VOC, posaconazole-POC, and itraconazole-ITC), polyenes (amphotericin B-AMB), and echinocandins (caspofungin-CAS) were tested. Of note, MIC (minimum inhibitory concentration) was defined as the lowest drug concentration that inhibits 50% of fungal cell growth in this study. Despite variations observed among different strains, we did not find any clinical isolates resistant to CAS or VOC. However, among the strains isolated from fermented food, we identified one strain resistant to CAS (PK-43, MIC = 1 mg/L) and one strain resistant to VOC (PK-27, MIC = 2 mg/L). Both strains (PK-43 and PK-27) were also relatively resistant to FLU (MIC value = 128 mg/L. Compared to clinical strains, a greater portion of *C. krusei* isolates from fermented foods exhibited resistant, intermediate, or non-WT strains to FLU, VOC, ITC, AMB, and CAS (Figure S3 and Table 1). However, this was not the case for POC. The clinical strains had a relatively higher average MIC to POC than the isolates from fermented foods (Figure S3). For example, 78.1% of isolates from fermented foods (25/32) were relatively resistant to FLU (MIC ≥ 64 mg/L), whereas only two clinical strains (2/54, 3.7%) were relatively resistant to FLU (MIC ≥ 64 mg/L) and 52 (52/54, 96.3%) relatively sensitive to this antifungal drug (MIC ≤ 32 mg/L). A similar pattern was observed in the MIC values for VOC and ITC. Among the FLU, VOC, ITC-resistant strains from clinical settings, a majority were isolated from sputum and urine samples. Among the non-WT clinical strains with an MIC ≥ 1 mg/L to POC, most were isolated from deep-seated infection, sputum, and urine samples (Table S1 and Dataset S1). Moreover, 2% (1/54) of the clinical isolates and 65.6% (21/32) of the isolates from fermented foods were non-WT for AMB (MIC ≥ 4 mg/L). 37.0% (20/54) of the clinical isolates and 3.1% (1/32) of the isolates from fermented foods were non-WT for POC (MIC ≥ 1 mg/L). 3.7% (2/54) of the clinical isolates and 18.8% (6/32) of the isolates from fermented foods were non-WT for ITC (MIC ≥ 2 mg/L). A summary of MIC values and detailed information on all antifungals tested are presented Table 1 and Supplementary Table S1, respectively. These findings raise the possibility of environmental origination of antifungal resistance in *C. krusei* (especially for azole resistance).

**Table 1.** Antifungal susceptibility for *C. krusei* isolates from clinical samples and fermented foods.

Source	Number of isolates at each determined MIC value											
	MIC (mg/L)*	≤ 0.125	0.25	0.5	1	2	4	8	16	32	64	≥128
Clinical sample	FLU	0	0	0	0	0	0	1	18	33	2	0
	AMB	0	0	0	21	32	0	1	0	0	0	0
	POC	2	10	22	20	0	0	0	0	0	0	0
	CAS	0	47	7	0	0	0	0	0	0	0	0
	VOC	0	48	6	0	0	0	0	0	0	0	0
	ITC	0	0	23	29	1	1	0	0	0	0	0
Fermented food	FLU	0	0	0	0	0	0	0	2	5	20	5
	AMB	0	0	0	0	11	21	0	0	0	0	0
	POC	0	18	13	0	1	0	0	0	0	0	0
	CAS	0	18	13	1	0	0	0	0	0	0	0
	VOC	0	15	7	9	1	0	0	0	0	0	0
	ITC	0	1	5	20	1	5	0	0	0	0	0

*54 clinical isolates and 32 fermented food isolates were examined. MIC, the minimum inhibitory concentration that inhibits the 50% of the growth compared to the drug-free control. FLU: Fluconazole, CAS: Caspofungin, AMB: Amphotericin B, ITC: Itraconazole, VOC: Voriconazole, POC: Posaconazole. Detailed information on antifungal susceptibility for each strain is presented in Table S1.

### Diploidy is prevalent in *C. krusei* clinical strains, whereas triploidy is prevalent in isolates from fermented foods

**Figure 2.**
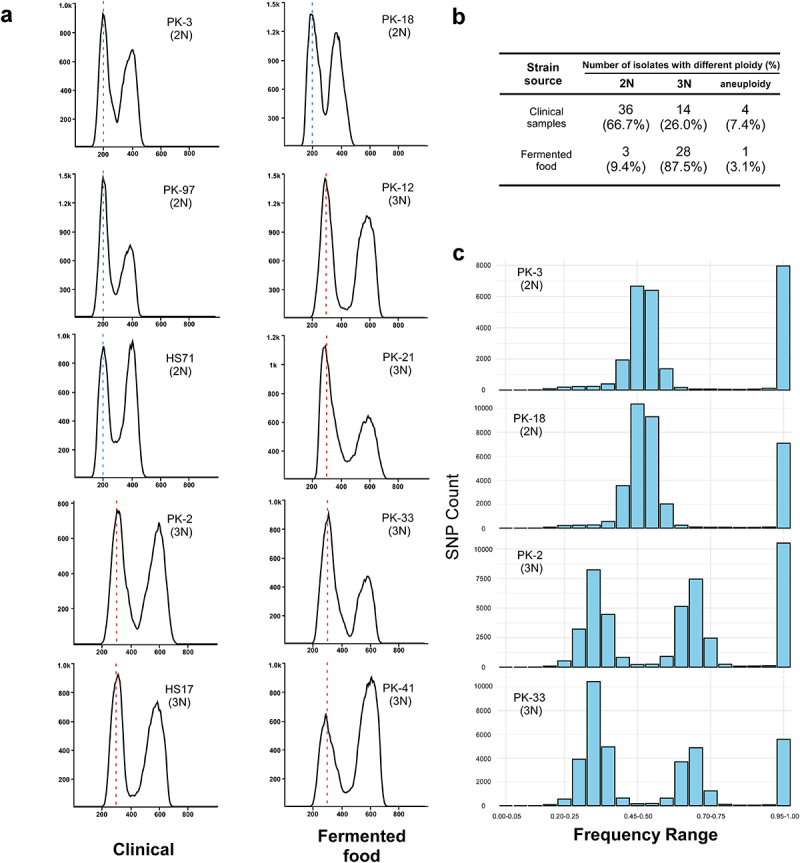
Analyses of ploidy and frequency of the non-reference allele for heterozygous biallelic SNPs. (a) FACS analysis of 10 representative *C. krusei* strains isolated from clinical samples and fermented foods. Fungal cells were stained with propidium iodide (PI) and subject to flow cytometry assays. The diploid FL2-H value at 200 is indicated by a light blue dashed line and the triploid FL2-H value at 300 is indicated by a red dashed line. (b) Numbers of diploid, triploid, and aneuploid isolates identified in clinical samples and fermented foods. Clinical samples: 24 from sputum samples or oral cavity, 10 from vaginal infections, 8 from urine samples, and 12 from deep-seated infections (blood, bile, abdominal drainage fluid, internal excreta, and ascites). (c) The frequency of the non-reference allele for heterozygous biallelic SNPs across all scaffolds in four representative isolates (PK-3, PK-18, PK-2, and PK-33). The Y-axis represents the number of SNPs, while the X-axis represents the non-reference allele frequency.

We noted that the cell size varied among different *C. krusei* strains in our cellular morphology analysis. Since cell size is often associated with ploidy in fungi [29,42–45], we performed flow cytometry assays to measure the genomic content of all 86 isolates we collected and the accuracy of flow cytometry results was confirmed through analysis of the frequency of the non-reference allele for heterozygous biallelic SNPs (Figure 2a, 2c, and Dataset S1). Of them, 94.2% of strains (*n* = 81) were euploid (diploid or triploid) and 5.8% (*n* = 5) were aneuploid (Figure 2b). To our surprise, 28 *C. krusei* isolates from fermented foods (28/32, 87.5%) had a triploid genome. One aneuploid and three diploid strains were found in the fermented food collection. However, only 14 clinical strains (14/54, 26.0%) were triploid and 4 clinical strains (4/54, 7.4%) were aneuploid (Figures 2b and S4). The aneuploid strains were verified using whole-genome sequencing assays (**Figure S5**). The majority of clinical strains (36/54, 66.7%) were diploid. These results suggest that ploidy variation of *C. krusei* strains could be an adaptive strategy to different ecological niches.

### Population structure analysis based on whole-genome SNPs

**Figure 3.**
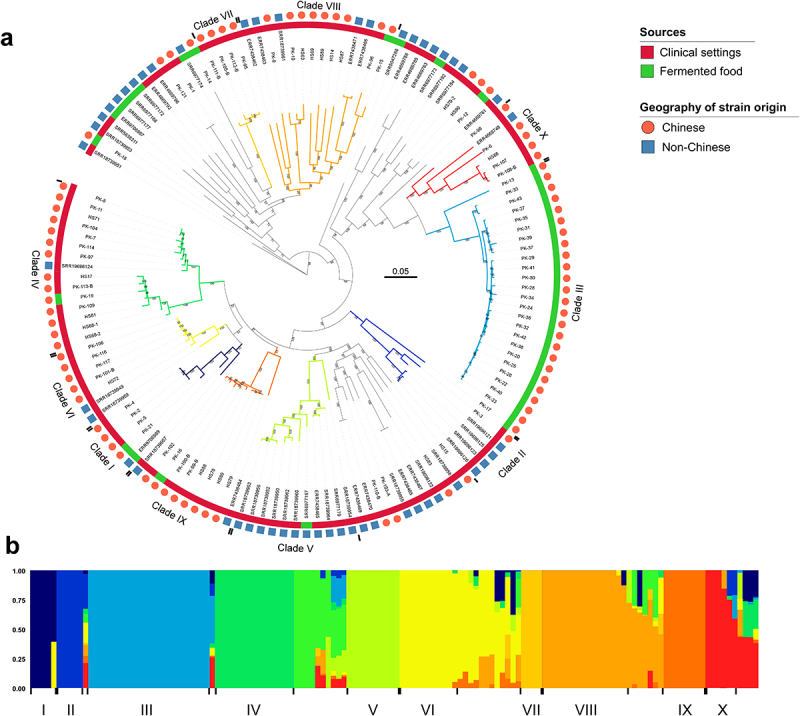
Phylogenetic and population structure analyses of *C. krusei* strains. Genomic data of 138 *C. krusei* isolates (86 strains sequenced in this study and 52 retrieved from the public available database) were used. (a) Maximum-likelihood phylogenetic tree based on 266,138 genome-wide SNPs. Scale bar, 0.05. The color strip displays the source of strains (red, clinical isolates; green, fermented food isolates). The outside squares and circles indicate the geographic origins of strains. Non-Chinese isolates were from the countries including Argentina, Denmark, Finland, Ghana, Hungary, Ireland, Italy, Japan, Poland, Russia, Singapore, USA, and Zimbabwe. (b) Population structure analysis. The ADMIXTURE program was used to perform population structure analysis based on the 266,138 genome-wide SNPs. The K (the number of populations assumed) value is set as 11 as determined by the minimum cross-validation error check.

**Figure 4.**
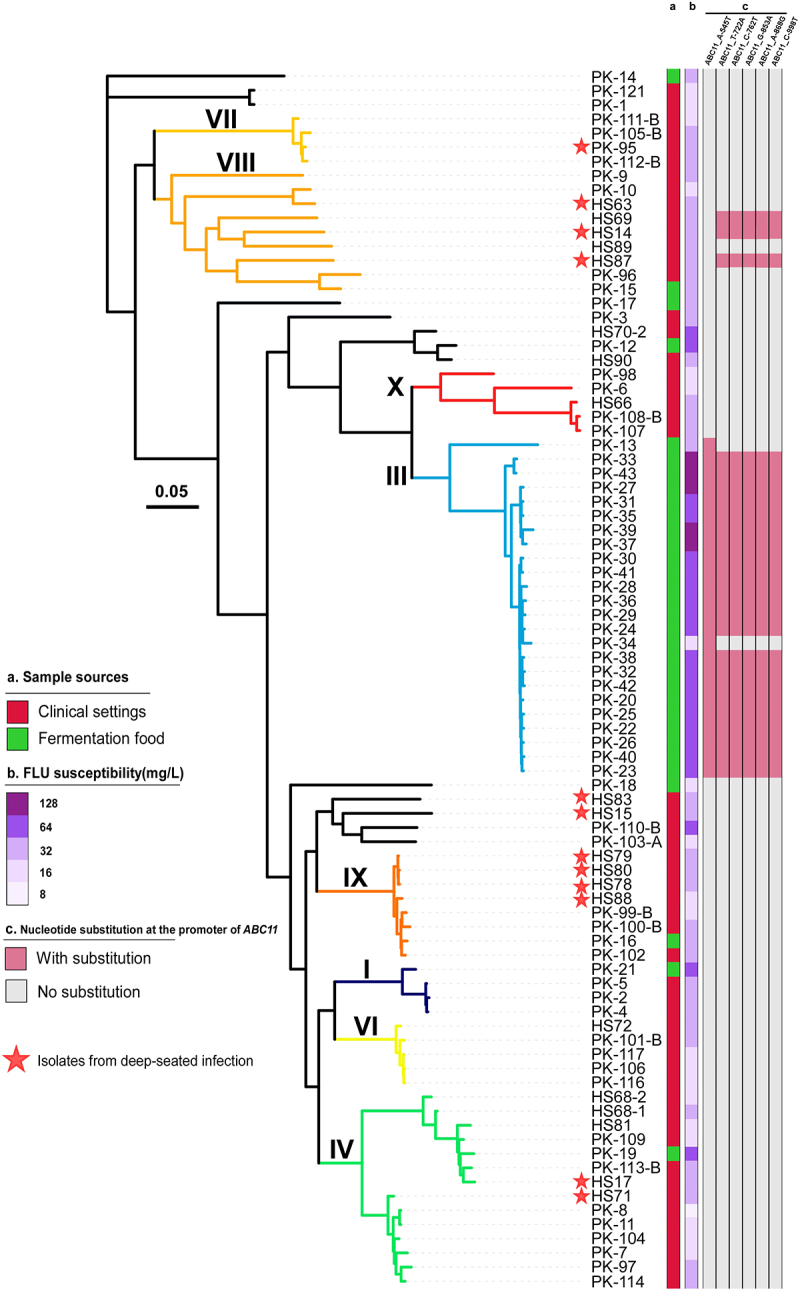
Phylogenetic analysis of *C. krusei* isolates from clinical samples and fermented foods. The maximum-likelihood tree was analyzed based on 185,429 genome-wide SNPs within 86 strains isolated in this study. The Bootstrap support value for each main clade was > 70%. (a) Sample sources. Red, clinical isolates; green, isolates from fermented foods. (b) Fluconazole (FLU) susceptibility indicated by the level of coloration. (c) Nucleotide substitutions at the promoter of *ABC11*. Pink, SNPs identified; grey, no SNP identified.

To explore genetic diversity and potential genetic association between *C. krusei* strains isolated from clinical settings and fermented foods, we performed whole-genome sequencing analysis and constructed phylogenetic trees. This analysis included the genomic sequences of all 86 isolates collected in this study (Chinese isolates) and 52 retrieved from the public database (non-Chinese isolates). In total, we identified 266,138 SNPs. Based on the topology of the tree and ADMIXTURE program analysis, we identified 10 genetic clades of *C. krusei* (Figure 3b). The topology of the phylogenetic tree containing only Chinese strains was highly similar to the tree including international strains (Figure 3a). The same clade numbering assay was used for the two phylogenetic trees. Based on these criteria, the Chinese strains collected in this study were distributed in eight clades (I, III, IV, and VI to X, Figure 4). Different degrees of genomic recombination were detected in 39.2% (38/97) of clinical isolates (Figure 3b). Interestingly, although isolates from fermented foods could be found in several genetic clades, a large subset of these strains were enriched in clade III, with no clinical isolates found in this clade. Population structure analysis demonstrated that some strains (e.g. a subset of strains from clade X) exhibited genomic recombination with clade III, indicating potential genetic communications among different clades (Figure 3b). Clinical strains were distributed in clades I, II, and IV to X (Figure 3a). Most of clinical strains isolated from different tissues or clinical samples were not clustered into certain genetic clades. However, a majority of the strains isolated from deep-seated infections (58.3%) belonged to clades VIII and IX. Most clades included strains isolated from both clinical settings and fermented foods, implying potential genetic association and strain transmissions between the two ecological niches.

To validate these findings, we constructed a maximum-likelihood phylogenetic tree of the Chinese *C. krusei* strains collected in this study based on 185,429 SNPs (Figure 4). The results of our maximum-likelihood phylogenetic analysis were consistent with those of the earlier assay (Figure 3).

Consistent with the result of flow cytometry assays, the genome-wide analysis of the frequency of heterozygous biallelic SNPs indicated that 94.2% (*n* = 81) of the Chinese *C. krusei* strains were euploid (diploid or triploid), while 5.8% (*n* = 5) of the strains were aneuploid (Figure 2, Dataset S1). Compared to the euploid strains, the aneuploid strains did not show an obvious increase in antifungal resistance. Interestingly, one aneuploid strain (PK-34, 2.7N) exhibited greater susceptibility to all antifungal drugs compared to the other strains in the same clade. However, the overall relative copy number of chromosome 4 in strain PK-34 was only 0.7. We noted that the azole drug resistance-associated gene *ABC11* is located at the proximal end of chromosome 4. Relative to the other strains of clade III, PK-34 showed a much lower MIC to FLU (16 mg/L). For clinical strains, no significant difference in antifungal susceptibility was found between diploid and triploid isolates. Most triploid strains (25/28, 89.3%) from fermented food exhibited an MIC ≥ 64 mg/L to FLU. It remains to be investigated whether the decreased susceptibility to FLU is associated to ploidy changes in the strains from fermented foods.

Based on the whole-genome sequencing analysis using the genomic sequence of strain CBS573 as the reference, we found that 59.3% (51/86) of strains exhibited an average heterozygosity of 0.43% and 40.7% (35/86) of strains had an average heterozygosity of 0.22% (Figure S6). The majority of these strains (83/86, 96.5%) were heterozygous at the mating type locus (*MTL* /α, //α or **a**/α/α, Figure S6). Three strains (3/86, 3.5%) were homozygous at the *MTL* locus (two α/α and one **a/a**, strains HS14, PK-96, and PK-7, Figures S5 and **S6**). Strains HS14 and PK-7 were diploid, while PK-96 was aneuploid.

**Figure 5.**
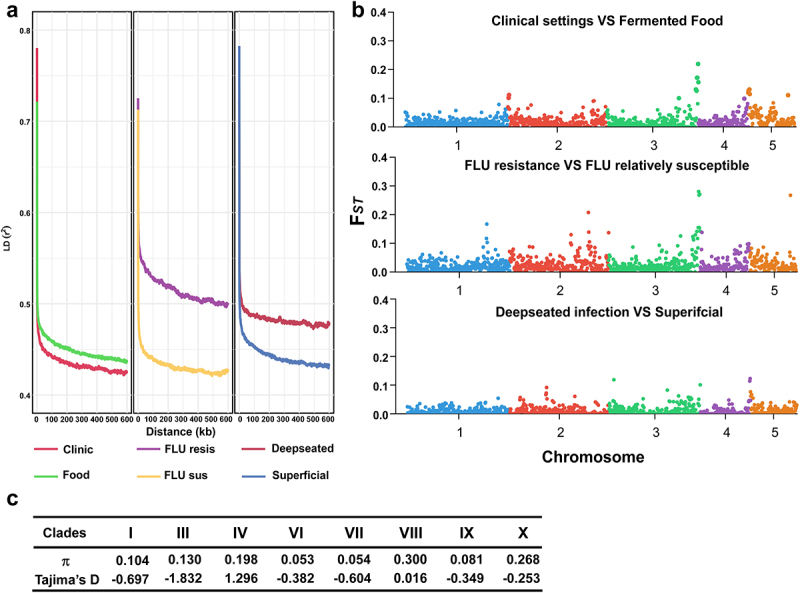
Genetic diversity analyses of *C. krusei* isolates from clinical samples and fermented foods. (a) Linkage disequilibrium decay analysis. Comparative analyses between isolates from clinical samples and fermented foods, fluconazole-resistant (flu-resis) and fluconazole-susceptible (flu-sus) isolates, and isolates from superficial (or colonizers) and deep-seated infections. The correlation coefficient (r^2^) for the first 600 kb of the genome was calculated using PLINK. Clinical isolates (*n* = 54), fermented food isolates (*n* = 32), flu-resistant (*n* = 25), flu-susceptible (*n* = 61), deep-seated (*n* = 10), and superficial (*n* = 76). (b) Genome-wide analysis of genetic divergence (F_*ST*_) using vcftools with non-overlapping 10 kb windows. Spots of different colors represent the windows on different chromosomes. (c) Tajima’s’s D and π values for each clade. Clade assignments were based on the genetic relationship between Chinese and international strains as well as the topological structure of the phylogenetic tree in figure 4.

To reveal the genetic diversity and association among different natural *C. krusei* isolates, we performed decay of linkage disequilibrium (LD) and genome-wide F_*ST*_ analyses. No obvious difference in the LD decay rates was observed between strains isolated from clinical settings and fermented foods (Figure 5a). However, the LD decay rate of the population of fluconazole-susceptible strains (MIC ≤ 32 mg/L) was faster than that of the fluconazole-resistant population (MIC ≥ 64 mg/L). Among clinical *C. krusei* isolates, the LD decay rate of the strain population from superficial infections was faster than that of the strains from deep-seated infections (Figure 5a). These results suggest that, compared to the drug-susceptible strains, the drug-resistant strains of *C. krusei* exhibited reduced genetic diversity, possibly due to the selection of antifungal stress. Similarly, the clinical isolates from deep-seated infections also showed decreased genetic diversity relatively to isolates from superficial samples. Genome-wide F_*ST*_ and nucleotide diversity (π) and Tajima’s D analyses further indicated a potential genetic association among strains from different ecological sources (Figure 5b, c).

### Nucleotide substitutions in the promoter region of the ABC11 are associated with fluconazole resistance

To explore the mechanism of antifungal resistance in natural *C. krusei* strains, we performed mutation analysis of drug resistance-related genes based on the whole-genome sequencing data. In the Chinese isolates, a homozygous missense amino acid mutation of Erg11 was found in only one strain (PK-103, Erg11^A15V^, 1/86, 1.2%), while heterozygous mutations of Erg11 were found in three strains (HS89^N82T^; PK-1^D226N^ and PK-121^D226N^). Moreover, amino acid mutations of Abc11 (77/86, 89.5%) and Mdr1 (80/86, 93.0%) were common.

Previous studies have reported that T642C and A756T point mutations in the ORF of *ERG11* could be associated with reduced susceptibility to itraconazole in *C. krusei* strains [36,37]. In our collection, we found that 22 clinical isolates (40.7%) and 27 strains from fermented vegetables (84.4%) carried the T642C substitution, while 10 clinical isolates (18.5%) and 4 strains from fermented vegetables (12.5%) contained the A756T substitution. We analyzed the association between the T642C and A756T substitutions and itraconazole resistance but did not find obvious correlations (p-value >0.1, *chi*-squared test).

**Figure 6.**
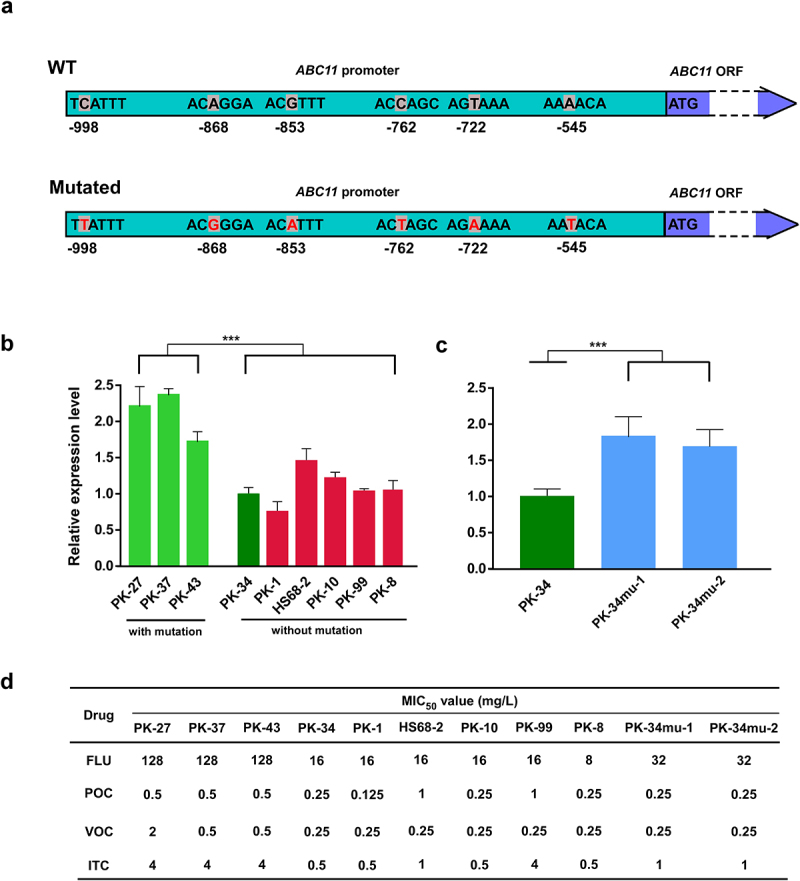
Verification of the effect of the five nucleotide substitutions in the promoter of *ABC11* on drug resistance. (a) Schematic diagram of the *ABC11* gene. The positions of nucleotide substitutions are indicated. (b) Relative expression levels of *ABC11* in the isolates with nucleotide substitutions (PK-27, PK-37, and PK-43; MIC of fluconazole >64  mg/L) and without nucleotide substitutions (PK-1, PK-8, PK-10, PK-99, and HS68-2; MIC of fluconazole < 32 mg/L). ***, indicates significant difference (*p* = 2.78e-06). (c) Relative expression levels of *ABC11* in strain PK-34 (without nucleotide substitutions) and re-constructed strains (PK-34mu-1 and PK-34mu-2) with nucleotide substitutions in the promoter of *ABC11*. A fragment carry the five nucleotide substitutions was cloned from strain PK-27 and introduced into strain PK-34, replacing the promoter region of one allele of *ABC11*. *** indicates a significant difference (*p* = 7.49e-05). (d) MIC of fluconazole (FLU), posaconazole (POC), voriconazole (VOC), and itraconazole (ITC). MIC, minimum inhibitory concentration required to inhibit the growth of 50% of fungal cells. All *p*-values were calculated based on two-tailed Student’s *t*-tests.

Notably, we identified six nucleotide substitutions of the *ABC11* promoter (A-545T, T-722A, C-762T, G-853A, A-868 G, and C-998T; Figure 6a) in clade III strains that were highly resistant to fluconazole (Figure 6a). Interestingly, one strain of clade III (PK-34) only carried one nucleotide substitution at the *ABC11* promoter region (A-545T). Consistently, compared to the other strains of clade III, this strain is relatively sensitive to FLU (with a MIC value = 16 mg/L). These findings suggest that the nucleotide substitutions in the *ABC11* promoter could be associated with the increased fluconazole resistance of clade III strains.

Quantitative RT-PCR assays demonstrated that the relative expression levels of *ABC11* in *C. krusei* strains carrying the six nucleotide substitutions were significantly higher than those in strains without mutations (clade III strain PK-34 and five strains from the other clades, Figure 6b). To further confirm the effect of the nucleotide substitutions at the *ABC11* promoter region on *ABC11* expression and fluconazole resistance, we replaced the promoter region of one *ABC11* allele in strain PK-34 with a copy of the fragment containing the six nucleotide substitutions (cloned from strain PK-27). Consistently, the two reconstituted strains (PK-34mu-1 and PK-34mu-2) exhibited an increased MIC to fluconazole and an increased relative expression level of *ABC11* (Figure 6d, c). Taken together, these results suggest that the nucleotide substitutions at the *ABC11* promoter region are associated with the increased fluconazole resistance of clade III strains.

## Discussion

C. krusei represents an emerging non-*albicans* pathogenic *Candida* species that can cause life-threatening infections in immunocompromised patients [6,46]. As a human commensal microbe, *C. krusei* can colonize the mucosal surface of healthy individuals, such as the digestive and respiratory tracts. Additionally, this yeast is widely distributed in natural environmental niches and is used in the food industry for fermentation [6,11]. It has been suggested that the use of azole fungicides in agriculture and food preservation could contribute to the development of antifungal resistance in human fungal pathogens [47,48]. In this study, we aimed to investigate the biological differences, genetic diversity, and associations between *C. krusei* strains isolated from clinical settings and traditional Chinese fermented food. Although the strains from these two sources exhibit distinct biological features, and a unique genetic clade (III) has been identified in the isolates from fermented foods, a large subset of *C. krusei* strains from clinical and environmental populations are genetically associated.*C. krusei* clinical strains generally exhibit a higher ability to undergo filamentation and biofilm development than isolates from fermented foods (Figures 1 and S1, Dataset S1). Interestingly, many *C. krusei* strains isolated from vaginal and deep-seated infections exhibited an enhanced ability for biofilm formation. Since morphological transitions and biofilm development are important virulence features of pathogenic fungi, this ability could represent a strategy of adaptation to the host niche [49]. We were unable to identify obvious genomic sequence changes between strains with distinct competences for filamentation or biofilm development. Further investigation is needed to determine whether genetic alterations or epigenetic changes contribute to this increased ability.

We also found that *C. krusei* strains from clinical settings and fermented foods showed distinct susceptibility to several antifungal drugs (Figure S3 and Table 1). Except for POC, *C. krusei* isolates from fermented foods were less susceptible to the other antifungal drugs we tested (FLU, VOC, ITC, AMB, and CAS) than clinical strains. This result was surprising. Further investigation is needed to determine whether azoles used in agriculture or for food preservation purposes contributed to this increased antifungal resistance in strains from fermented foods. Recent studies have demonstrated that environmental *Candida tropicalis* isolates from soil, beaches, and fruits could be reservoirs of resistant strains and could be transmitted to clinical settings [50,51]. Antifungal resistance could develop in these species during their life cycle in environmental niches. Of note, although the MIC value to CAS varied among different *C. krusei* isolates, no previously reported hotspot mutations of *FSK1* [26] were found in all isolates tested in this study perhaps due to the relatively low MIC value to CAS (≤1 mg/L).

*C. krusei* has been thought to be a diploid organism [6]. Triploid *C. krusei* strains have also been observed previously [11]. Interestingly, we found that a majority of *C. krusei* strains were isolated from fermented vegetables and one-third of clinical strains were triploid (Figure 2 and Dataset S1). The triploid form is more prevalent in environmental and clinical settings than previously thought. It remains unknown why triploid *C. krusei* strains are so prevalent in fermented vegetables. It remains to be investigated whether environmental stresses, such as salty and nutrient stresses of the fermented vegetables, could promote the formation of triploidy. In turn, the triploid form could obtain an advantage to survive and grow under such ecological niches. All fluconazole relatively resistant strains (25/25) isolated fermented vegetables were triploid, suggesting that increased ploidy may be a potential mechanism for fluconazole resistance in environmental *C. krusei* strains. However, this was not the case for clinical strains. No obvious association between triploidy formation and antifungal resistance was observed in clinical *C. krusei* strains.

The intrinsic fluconazole resistance of *C. krusei* poses a threat to human health. Although we did not find any “hotspot mutations” of the *ERG11* gene, which encodes the fluconazole target (lanosterol 14α-demethylase), genomic sequencing analysis identified several nucleotide substitutions in the promoter region of the *ABC11* gene. Since the Abc11 protein is critical for drug pump and resistance in fungi [52], these nucleotide substitutions could lead to the altered expression level of *ABC11* and decreased susceptibility to azoles in *C. krusei*. By introducing these mutations into a “wild type” strain, we confirmed that the nucleotide substitutions indeed play a critical role in the regulation of *ABC11* and fluconazole resistance (Figure 6c), which may represent a new mechanism of antifungal resistance in *C. krusei*.

Many human fungal pathogens, such as *C. krusei* and *C. tropicalis*, are widely distributed in the natural environment. Fungicides (especially azole antifungals) used in agriculture and food biopreservation could promote the development of antifungal resistance in these species. The spread of drug-resistant strains to clinical settings could lead to the failure of antifungal treatment. Our study demonstrates that drug-resistant *C. krusei* strains ubiquitously exist in traditional fermented vegetables and are genetically associated with clinical isolates. However, the current study has some limitations. First, the strain sample sizes of both the environmental and clinical sources were relatively small. Second, only a few of clinical strains were isolated from blood or deep-seated infections. Third, all strain samples were isolated in China and *C. krusei* isolated from other countries should be investigated in future research. Given the worldwide increasing incidence of *C. krusei*-caused infections, a One Health approach will be needed to combat the spread of this pathogen and ensure successful treatments.

## Supplementary Material

Figure S1.jpg

Dataset S1 Information for All Isolates0705.xlsx

Dataset S2 Primer information0705.xlsx

Figure S3.jpg

Figure S2.jpg

Figure S4.jpg

Figure S6.jpg

Figure S5.jpg

Table S1 Summary of Candida krusei antifungal susceptibility data.docx

## Data Availability

The Genomic data has been deposited in the NCBI database (https://www.ncbi.nlm.nih.gov/bioproject/PRJNA1101673). The data that support the findings of this study are available in Figshare at http://doi.org/10.6084/m9.figshare.25804882.
