# Supplementary figures and images for: Biology and genetic diversity of Candida krusei isolates from fermented vegetables and clinical samples in China
Source: Virulence. 2024 Oct 2;15(1):2411543. doi: 10.1080/21505594.2024.2411543 (PMC11487970; doi:10.1080/21505594.2024.2411543)

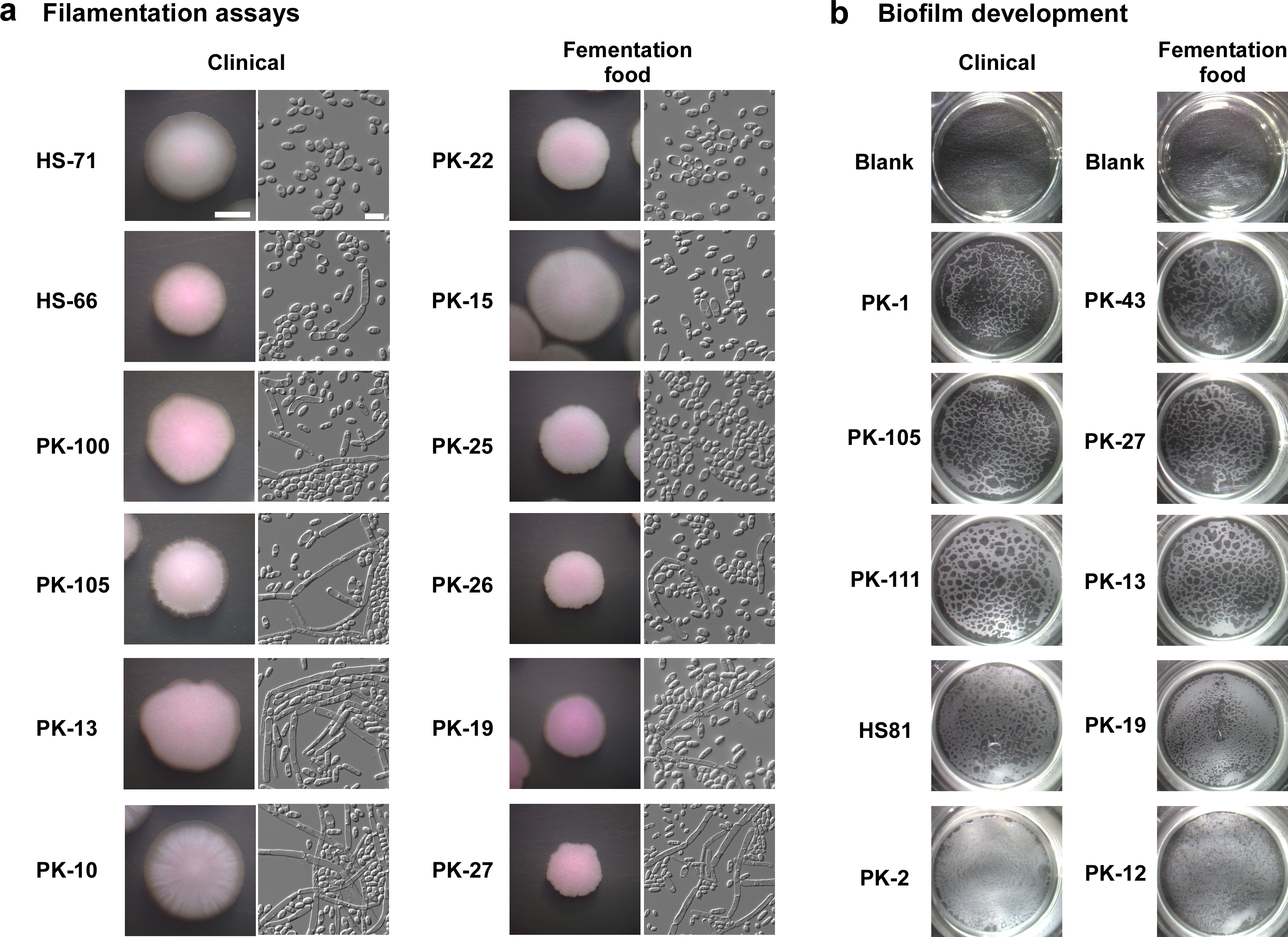

Supplement: Figure S1.jpg [file KVIR_A_2411543_SM0279.jpg]

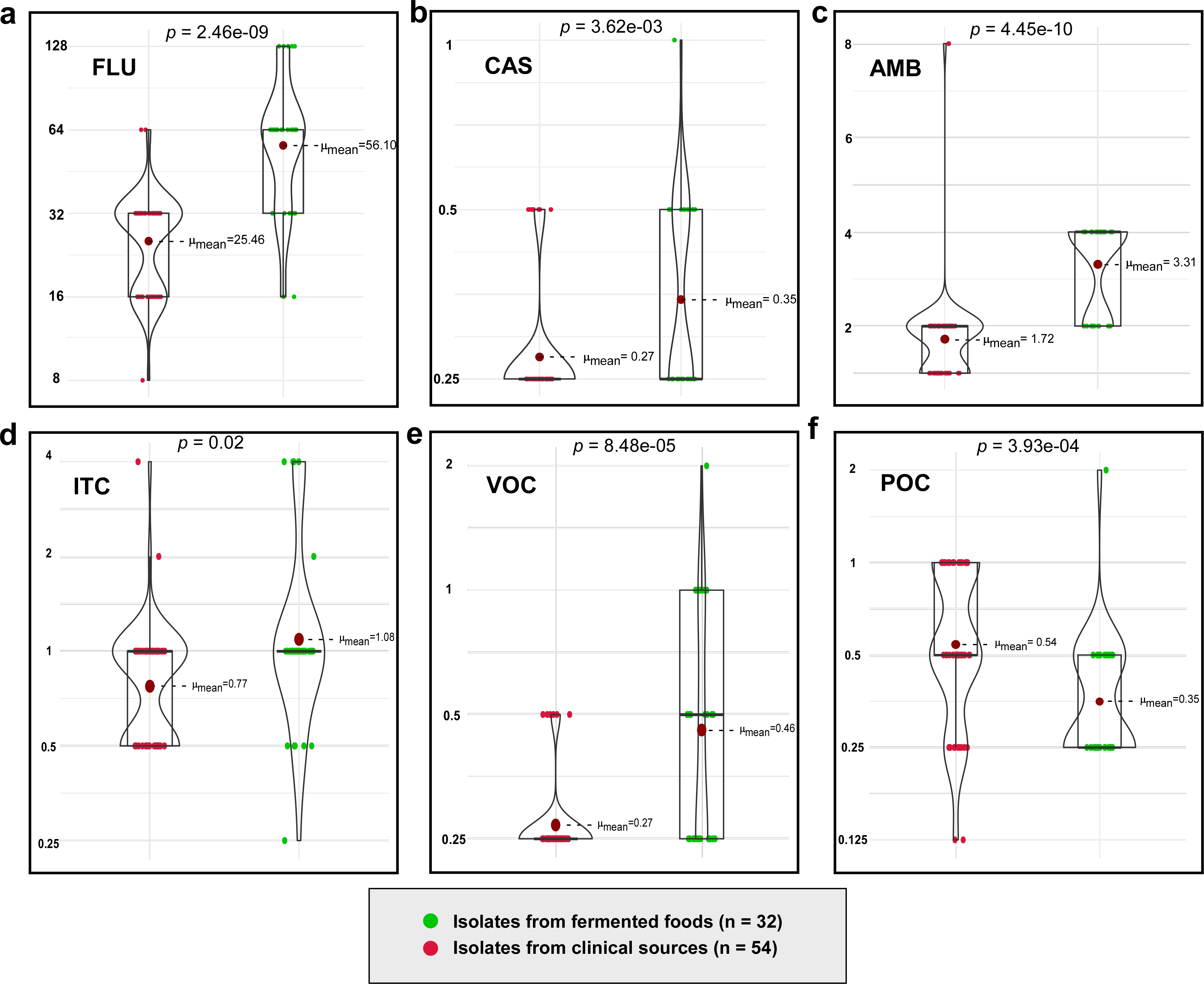

Supplement: Figure S3.jpg [file KVIR_A_2411543_SM0276.jpg]

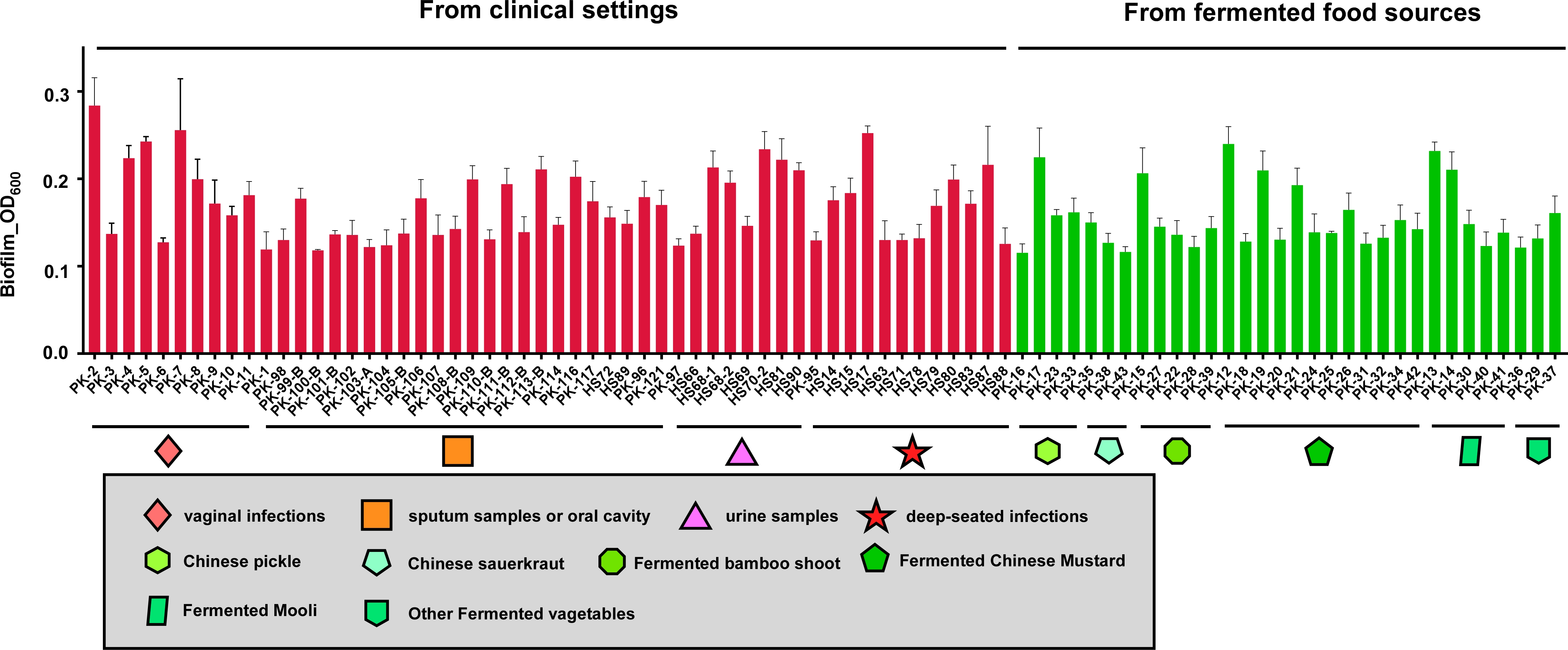

Supplement: Figure S2.jpg [file KVIR_A_2411543_SM0275.jpg]

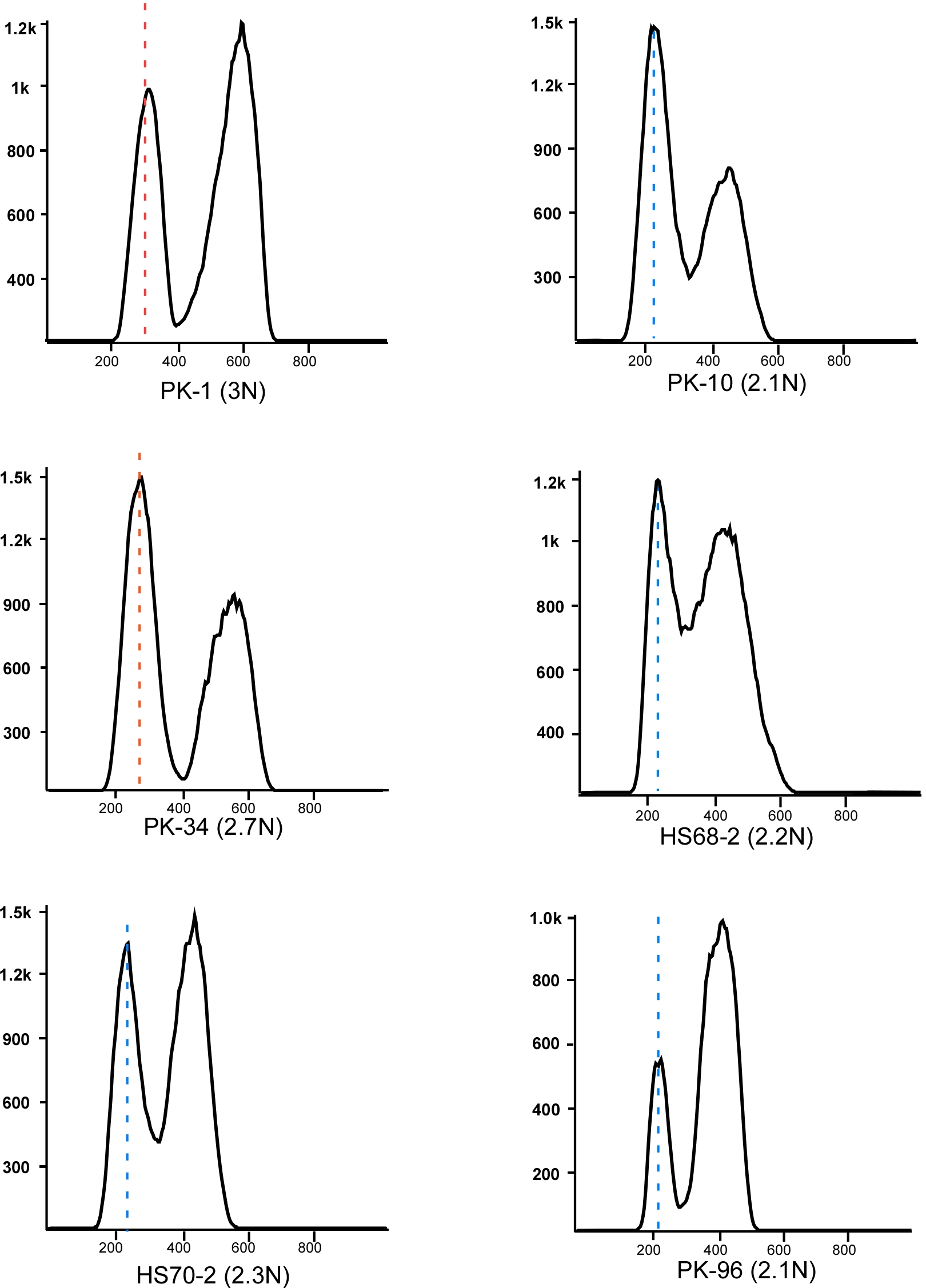

Supplement: Figure S4.jpg [file KVIR_A_2411543_SM0274.jpg]

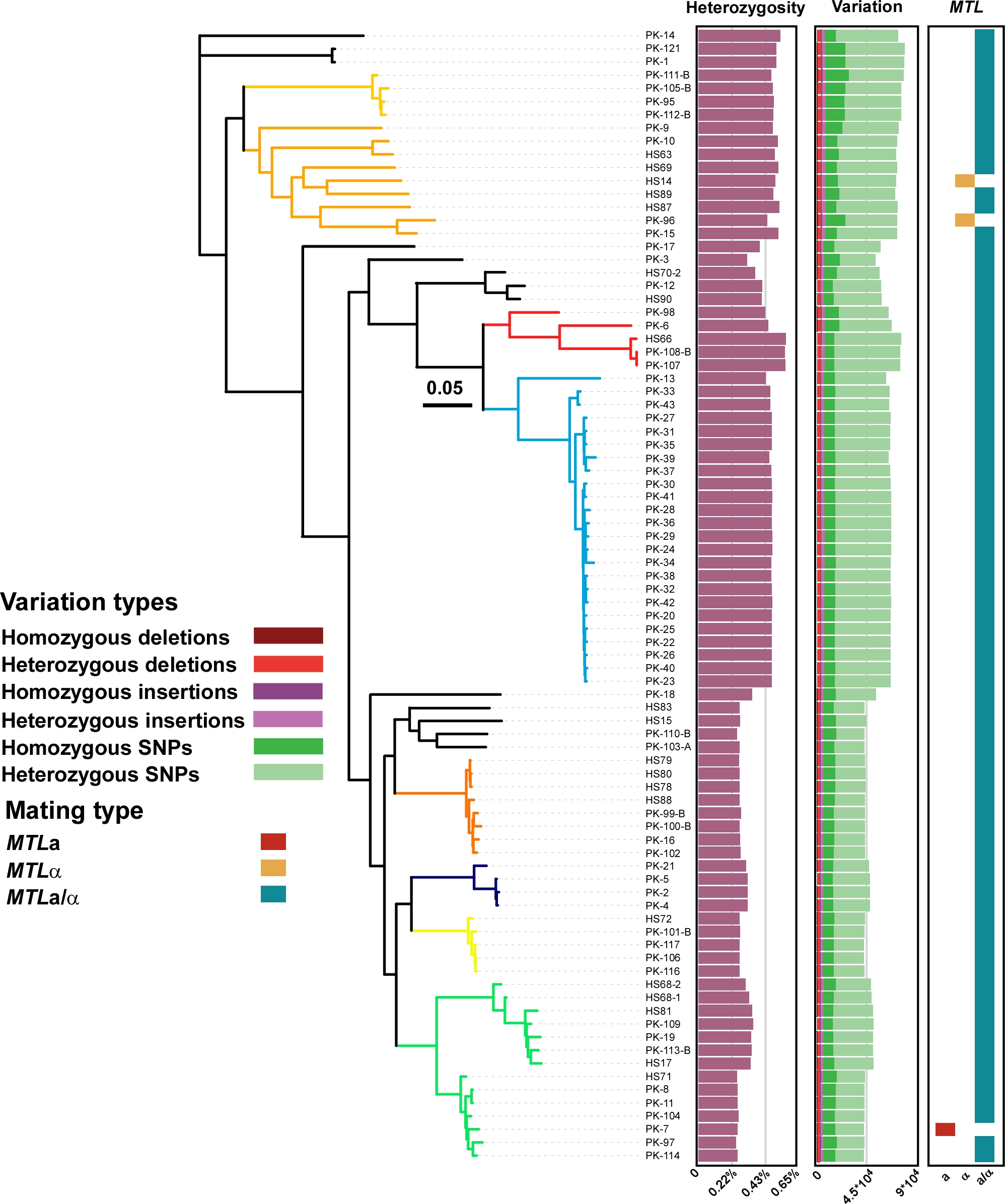

Supplement: Figure S6.jpg [file KVIR_A_2411543_SM0273.jpg]

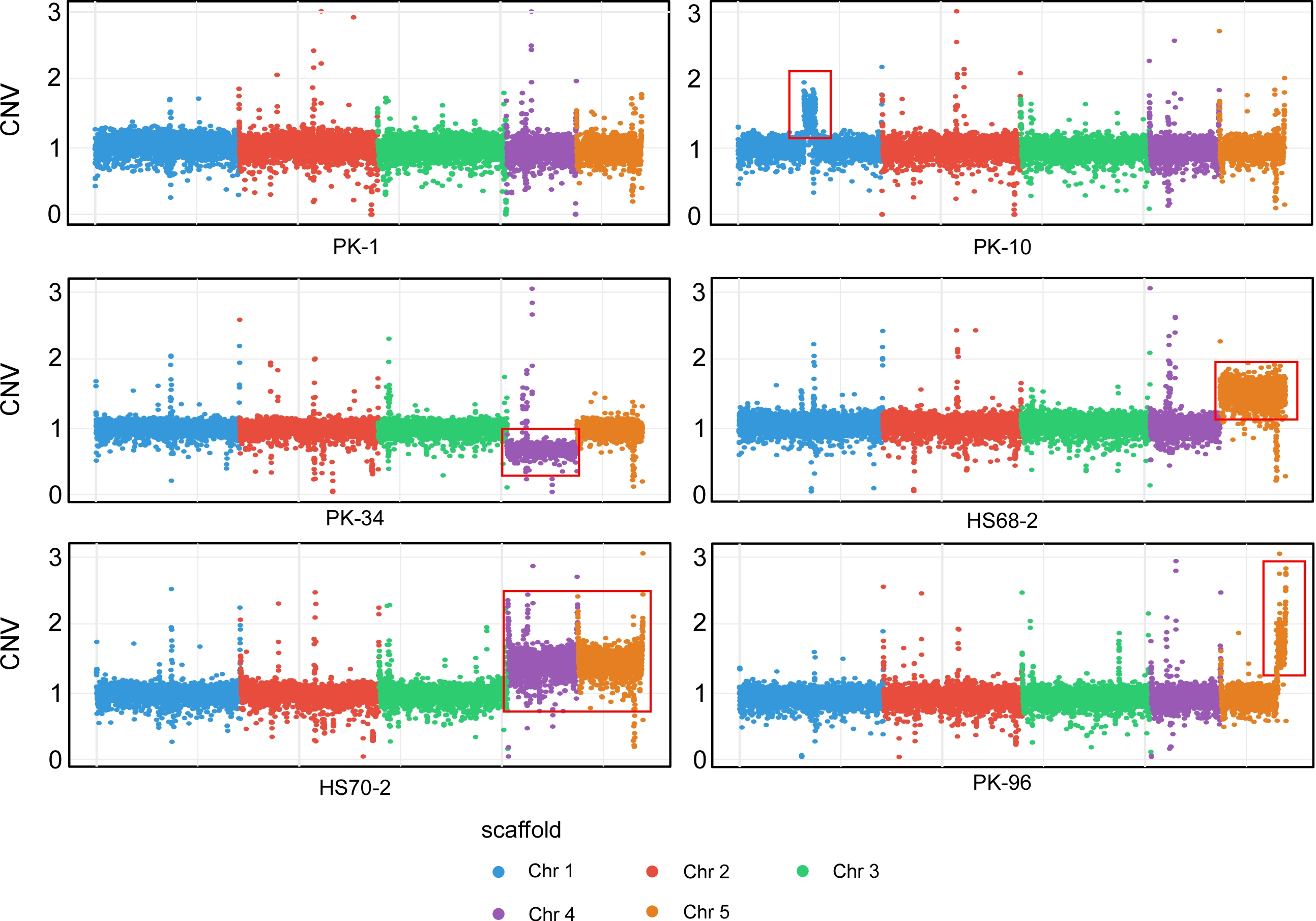

Supplement: Figure S5.jpg [file KVIR_A_2411543_SM0272.jpg]
