# Supplementary material for: Biology and genetic diversity of Candida krusei isolates from fermented vegetables and clinical samples in China
Source: Virulence. 2024 Oct 2;15(1):2411543. doi: 10.1080/21505594.2024.2411543 (PMC11487970; doi:10.1080/21505594.2024.2411543)
Supplement: Table S1 Summary of Candida krusei antifungal susceptibility data.docx [file KVIR_A_2411543_SM0271.docx]

**Table S1. Summary of *Candida krusei* antifungal susceptibility data**

|  |  | **MIC (mg/L)** | | | | | |
| --- | --- | --- | --- | --- | --- | --- | --- |
| **Source** | **Isolates** | **FLU** | **CAS** | **AMB** | **ITC** | **VOC** | **POC** |
| ***C. krusei***  **from**  **clinical samples** | PK-1 | 16 | 0.25 | 2 | 0.5 | 0.25 | 0.125 |
|  | PK-2 | 32 | 0.25 | 2 | 0.5 | 0.25 | 0.25 |
|  | PK-3 | 32 | 0.5 | 2 | 0.5 | 0.25 | 0.25 |
|  | PK-4 | 32 | 0.25 | 2 | 0.5 | 0.25 | 0.25 |
|  | PK-5 | 32 | 0.25 | 2 | 0.5 | 0.25 | 0.25 |
|  | PK-6 | 16 | 0.25 | 2 | 0.5 | 0.25 | 0.25 |
|  | PK-7 | 16 | 0.25 | 2 | 0.5 | 0.25 | 0.25 |
|  | PK-8 | 8 | 0.25 | 2 | 0.5 | 0.25 | 0.25 |
|  | PK-9 | 32 | 0.25 | 2 | 0.5 | 0.25 | 0.25 |
|  | PK-10 | 16 | 0.25 | 2 | 0.5 | 0.25 | 0.25 |
|  | PK-11 | 16 | 0.25 | 2 | 0.5 | 0.25 | 0.25 |
|  | PK-95 | 32 | 0.25 | 2 | 1 | 0.25 | 0.5 |
|  | PK-96 | 32 | 0.25 | 1 | 1 | 0.25 | 1 |
|  | PK-97 | 32 | 0.25 | 2 | 1 | 0.25 | 0.5 |
|  | PK-98 | 16 | 0.25 | 2 | 2 | 0.25 | 0.5 |
|  | PK-99 | 16 | 0.25 | 2 | 4 | 0.25 | 1 |
|  | PK-100 | 32 | 0.25 | 2 | 1 | 0.25 | 0.5 |
|  | PK-101 | 32 | 0.25 | 2 | 1 | 0.5 | 0.5 |
|  | PK-102 | 32 | 0.25 | 2 | 1 | 0.5 | 1 |
|  | PK-103 | 16 | 0.25 | 2 | 1 | 0.25 | 0.5 |
|  | PK-104 | 16 | 0.25 | 2 | 1 | 0.25 | 0.5 |
|  | PK-105 | 32 | 0.25 | 8 | 1 | 0.25 | 1 |
|  | PK-106 | 16 | 0.25 | 2 | 0.5 | 0.25 | 0.5 |
|  | PK-107 | 32 | 0.25 | 2 | 1 | 0.25 | 0.5 |
|  | PK-108 | 32 | 0.25 | 2 | 1 | 0.25 | 1 |
|  | PK-109 | 16 | 0.25 | 2 | 1 | 0.25 | 1 |
|  | PK-110 | 64 | 0.25 | 2 | 1 | 0.5 | 1 |
|  | PK-111 | 16 | 0.25 | 2 | 1 | 0.25 | 0.5 |
|  | PK-112 | 32 | 0.5 | 2 | 1 | 0.25 | 0.5 |
|  | PK-113 | 32 | 0.25 | 2 | 1 | 0.25 | 0.5 |
|  | PK-114 | 32 | 0.25 | 2 | 1 | 0.25 | 1 |
|  | PK-116 | 16 | 0.5 | 2 | 1 | 0.25 | 0.5 |
|  | PK-117 | 16 | 0.5 | 2 | 1 | 0.25 | 0.5 |
|  | PK-121 | 16 | 0.25 | 2 | 0.5 | 0.25 | 0.125 |
|  | HS14 | 32 | 0.25 | 1 | 0.5 | 0.25 | 0.5 |
|  | HS15 | 32 | 0.5 | 1 | 0.5 | 0.25 | 1 |
|  | HS17 | 32 | 0.25 | 1 | 1 | 0.25 | 1 |
| ***C. krusei***  **from**  **clinical samples** | HS90 | 32 | 0.25 | 1 | 0.5 | 0.25 | 1 |
|  | HS63 | 32 | 0.5 | 1 | 1 | 0.5 | 1 |
|  | HS66 | 32 | 0.25 | 1 | 0.5 | 0.25 | 1 |
|  | HS68-1 | 32 | 0.25 | 1 | 1 | 0.25 | 1 |
|  | HS68-2 | 16 | 0.25 | 1 | 1 | 0.25 | 1 |
|  | HS69 | 32 | 0.25 | 1 | 1 | 0.25 | 1 |
|  | HS70-2 | 64 | 0.25 | 1 | 1 | 0.5 | 1 |
|  | HS71 | 32 | 0.25 | 1 | 1 | 0.25 | 0.5 |
|  | HS72 | 32 | 0.25 | 1 | 0.5 | 0.25 | 1 |
|  | HS78 | 32 | 0.25 | 1 | 0.5 | 0.25 | 0.5 |
|  | HS79 | 32 | 0.25 | 1 | 0.5 | 0.25 | 0.5 |
|  | HS80 | 32 | 0.25 | 1 | 0.5 | 0.25 | 0.5 |
|  | HS81 | 16 | 0.25 | 1 | 0.5 | 0.25 | 0.5 |
|  | HS83 | 32 | 0.5 | 1 | 1 | 0.25 | 0.5 |
|  | HS87 | 32 | 0.25 | 1 | 1 | 0.25 | 1 |
|  | HS88 | 16 | 0.25 | 1 | 0.5 | 0.25 | 0.5 |
|  | HS89 | 32 | 0.25 | 1 | 1 | 0.5 | 1 |
| ***C. krusei***  **from**  **Fermented foods**  ***C. krusei***  **from**  **Fermented foods** | PK-12 | 64 | 0.5 | 4 | 0.5 | 0.25 | 0.25 |
|  | PK-13 | 32 | 0.5 | 4 | 0.5 | 0.25 | 0.25 |
|  | PK-14 | 32 | 0.5 | 4 | 0.5 | 0.25 | 0.25 |
|  | PK-15 | 32 | 0.5 | 4 | 1 | 0.25 | 0.25 |
|  | PK-16 | 32 | 0.25 | 4 | 1 | 0.25 | 0.25 |
|  | PK-17 | 32 | 0.25 | 4 | 0.5 | 0.25 | 0.25 |
|  | PK-18 | 16 | 0.25 | 4 | 1 | 1 | 0.25 |
|  | PK-19 | 64 | 0.25 | 4 | 0.25 | 0.25 | 0.25 |
|  | PK-20 | 64 | 0.25 | 4 | 1 | 1 | 0.25 |
|  | PK-21 | 64 | 0.25 | 4 | 1 | 1 | 0.25 |
|  | PK-22 | 64 | 0.25 | 4 | 1 | 1 | 0.25 |
|  | PK-23 | 64 | 0.25 | 4 | 1 | 1 | 0.25 |
|  | PK-24 | 64 | 0.25 | 4 | 1 | 1 | 0.25 |
|  | PK-25 | 64 | 0.5 | 4 | 1 | 1 | 0.5 |
|  | PK-26 | 64 | 0.25 | 4 | 1 | 1 | 0.25 |
|  | PK-27 | 128 | 0.5 | 2 | 4 | 2 | 0.5 |
|  | PK-28 | 64 | 0.25 | 4 | 1 | 1 | 0.25 |
|  | PK-29 | 64 | 0.25 | 4 | 1 | 0.25 | 0.25 |
|  | PK-30 | 64 | 0.25 | 2 | 1 | 0.5 | 0.5 |
|  | PK-31 | 64 | 0.5 | 2 | 2 | 0.25 | 0.5 |
|  | PK-32 | 64 | 0.25 | 2 | 1 | 0.25 | 0.5 |
|  | PK-33 | 128 | 0.5 | 2 | 4 | 0.5 | 2 |
|  | PK-34 | 16 | 0.25 | 2 | 0.5 | 0.25 | 0.25 |
|  | PK-35 | 64 | 0.25 | 2 | 1 | 0.5 | 0.5 |
|  | PK-36 | 64 | 0.5 | 4 | 1 | 0.25 | 0.25 |
|  | PK-37 | 128 | 0.5 | 4 | 4 | 0.5 | 0.5 |
|  | PK-38 | 64 | 0.25 | 2 | 1 | 0.25 | 0.5 |
|  | PK-39 | 128 | 0.5 | 2 | 4 | 0.5 | 0.5 |
|  | PK-40 | 64 | 0.25 | 4 | 1 | 0.25 | 0.5 |
|  | PK-41 | 64 | 0.5 | 2 | 1 | 0.25 | 0.5 |
|  | PK-42 | 64 | 0.5 | 2 | 1 | 0.5 | 0.5 |
|  | PK-43 | 128 | 1 | 4 | 4 | 0.5 | 0.5 |

FLU:Fluconazole, CAS: Caspofungin, AMB: Amphotericin B, ITC: Itraconazole, VOC: Voriconazole, POC: Posaconazole
